# A Novel Strategy for Development of Recombinant Antitoxin Therapeutics Tested in a Mouse Botulism Model

**DOI:** 10.1371/journal.pone.0029941

**Published:** 2012-01-06

**Authors:** Jean Mukherjee, Jacqueline M. Tremblay, Clinton E. Leysath, Kwasi Ofori, Karen Baldwin, Xiaochuan Feng, Daniela Bedenice, Robert P. Webb, Patrick M. Wright, Leonard A. Smith, Saul Tzipori, Charles B. Shoemaker

**Affiliations:** 1 Department of Biomedical Sciences, Tufts Cummings School of Veterinary Medicine, North Grafton, Massachusetts, United States of America; 2 National Institute of Allergy and Infectious Diseases, National Institutes of Health, Bethesda, Maryland, United States of America; 3 United States Army Medical Research Institute for Infectious Diseases, Frederick, Maryland, United States of America; University of Wisconsin, Food Research Institute, United States of America

## Abstract

Antitoxins are needed that can be produced economically with improved safety and shelf life compared to conventional antisera-based therapeutics. Here we report a practical strategy for development of simple antitoxin therapeutics with substantial advantages over currently available treatments. The therapeutic strategy employs a single recombinant ‘targeting agent’ that binds a toxin at two unique sites and a ‘clearing Ab’ that binds two epitopes present on each targeting agent. Co-administration of the targeting agent and the clearing Ab results in decoration of the toxin with up to four Abs to promote accelerated clearance. The therapeutic strategy was applied to two *Botulinum* neurotoxin (BoNT) serotypes and protected mice from lethality in two different intoxication models with an efficacy equivalent to conventional antitoxin serum. Targeting agents were a single recombinant protein consisting of a heterodimer of two camelid anti-BoNT heavy-chain-only Ab V_H_ (VHH) binding domains and two E-tag epitopes. The clearing mAb was an anti-E-tag mAb. By comparing the in vivo efficacy of treatments that employed neutralizing vs. non-neutralizing agents or the presence vs. absence of clearing Ab permitted unprecedented insight into the roles of toxin neutralization and clearance in antitoxin efficacy. Surprisingly, when a post-intoxication treatment model was used, a toxin-neutralizing heterodimer agent fully protected mice from intoxication even in the absence of clearing Ab. Thus a single, easy-to-produce recombinant protein was as efficacious as polyclonal antiserum in a clinically-relevant mouse model of botulism. This strategy should have widespread application in antitoxin development and other therapies in which neutralization and/or accelerated clearance of a serum biomolecule can offer therapeutic benefit.

## Introduction

The presence of toxins in circulation is the cause of a wide variety of human and animal illnesses. Antitoxins are therapeutic agents that reduce further development of symptoms in patients that have been exposed to a toxin. Typically, antitoxins are the antisera obtained from large animals that were immunized with inactivated toxin [1], [2]. More recently, some antitoxin therapies have been developed using one or more antitoxin mAbs [3], [4], [5], [6]. Antisera and mAbs can be difficult to produce economically at scale, usually require long development times and often have problematic quality control, shelf-life and safety issues. New therapeutic strategies to develop and prepare antitoxins are needed.

Antitoxins function through two key mechanisms; neutralization of toxin function and clearance of toxin from the body. Toxin neutralization can occur through processes such as inhibition of enzymatic activity and prevention of binding to cellular receptors. Antibody mediated clearance from serum is thought to occur subsequent to the binding of multiple antibodies to the target antigen [7], [8], [9], [10]. Multimeric antibody decoration of the target is considered necessary to permit binding to low affinity Fc receptors [8], [10]. An ideal antitoxin therapeutic will both promote toxin neutralization to immediately block further toxin activity and accelerate toxin clearance to eliminate future pathology if neutralization becomes reversed.

*Clostridium botulinum* neurotoxin (BoNT) is a National Institute of Allergy and Infectious Diseases (NIAID) Category A priority pathogen which can cause botulism, a potentially lethal flaccid paralysis. Currently, the only treatments for botulism are antitoxins. Polyclonal antitoxin sera are available to treat infants (BabyBIG [11]) or adults (HBAT [12]) that become exposed to BoNT and these can prevent further development of paralysis. Once serious paralysis has occurred, though, palliative care is the only available option [13]. Some laboratories are working to develop monoclonal antibodies (mAbs) as possible antitoxin alternatives to polyclonal antisera [3], [14], [15], [16], [17]. Nowakowski et al [3] found that effective protection of mice against high dose challenge of BoNT serotype A (BoNT/A) required co-administration of three antitoxin mAbs, presumably to promote clearance. We previously demonstrated that administration of a pool of three or more small binding agents, each produced with a common epitopic tag, dramatically reduced serum levels of a toxin when co-administered with an anti-tag mAb [18]. The tagged binding agents directed the binding of anti-tag mAb to multiple sites on the toxin, thus indirectly decorating the toxin with Ab Fc domains and leading to its clearance through the liver.

The use of small binding agents to direct the decoration of toxin with Ab permits new strategies for the development of agents with improved commercial properties. One binding agent scaffold with excellent properties is the camelid heavy-chain-only Ab V_H_ (VHH) domain. VHHs are small (∼12 kD), easy to produce, and generally more stable than conventional antibody fragments [19], [20]. They are often found to have unusual epitope specificities, particularly an improved ability to bind active site pockets to produce enzyme inhibition [21]. Because of the many favourable properties of VHHs, they have become widely used in research and show clear commercial potential [22], [23].

Here we show that a single recombinant heterodimeric binding agent consisting of two high-affinity BoNT binding VHH agents and two epitopic tags, co-administered with an anti-tag mAb, protected mice from lethality with an efficacy equivalent to conventional BoNT antitoxin serum in two different in vivo assays. Studies comparing neutralizing or non-neutralizing binding agents administered with or without clearing Ab provide a unique method for evaluating the relative contributions of toxin neutralization and toxin clearance to antitoxin efficacy. We show that toxin neutralization and toxin clearance both contribute significantly to antitoxin efficacy in mice. Using the heterodimer antitoxin strategy, toxin neutralization or toxin clearance alone proved to be sufficient to protect mice from BoNT intoxication in a therapeutically relevant, post-intoxication assay.

## Results

### Identification and characterization of anti-BoNT VHHs

Serum clearance of the protein, *Botulinum* neurotoxin serotype A (BoNT/A), can be dramatically accelerated by administering a pool of different epitopically-tagged single-chain Ig variable fragment (scFv) domain binding agents together with an anti-tag mAb [18]. To determine whether similar results could be obtained using a more commercially and clinically acceptable binding agent, a panel of camelid heavy-chain-only Vh (VHH) binding agents were obtained having high affinity for BoNT/A holotoxin. In addition, VHHs were obtained that bind to BoNT serotype B (BoNT/B) holotoxin to permit efficacy testing on a second pathogenic serum protein. Competition ELISAs were used to identify the VHHs with the highest apparent affinity for unique epitopes on BoNT/A and BoNT/B leading to the selection of seven BoNT/A VHHs (Figure S1A) and four BoNT/B VHHs (Figure S1B). Each VHH was purified from *E. coli* as a thioredoxin fusion protein containing a single carboxyl-terminal epitopic tag (E-tag) (SDS-PAGE shown in Figure S2A).

**Figure 1:**
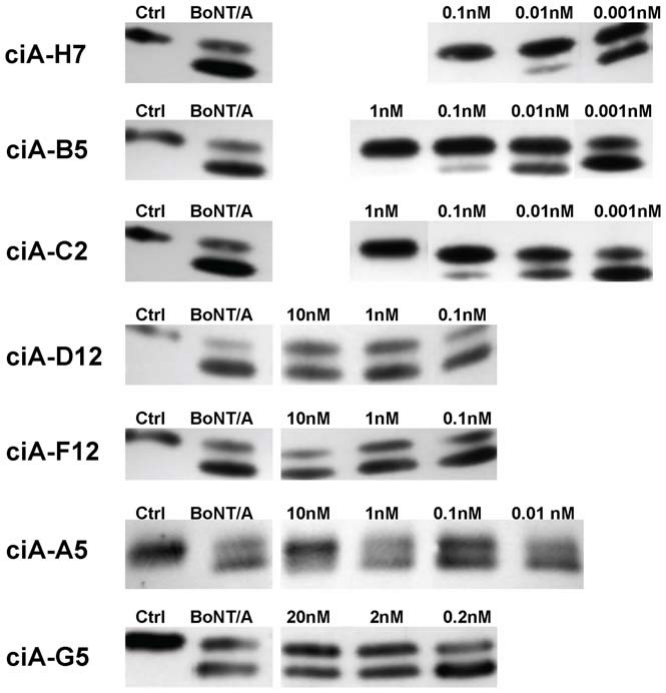
VHH neutralization of BoNT/A in rat primary cerebellar neuron cultures. Purified anti-BoNT/A VHHs (ciA-) were added to the medium for cultured primary cerebellar neurons at the indicated concentrations and then BoNT/A (∼10 pM) or medium (Ctrl) was added. After overnight culture, the cells were harvested and the extent of SNAP25 cleavage was assessed by Western blot. The upper band represents uncleaved SNAP25 while the lower band is BoNT/A cleaved SNAP25. VHHs that clearly inhibited BoNT/A cleavage of SNAP25 at concentrations of 0.1 nM were considered to be strong neutralizing agents (Table 1). Data shown for each VHH were obtained within the same experiment and are representative of at least two independent experiments.

The seven unique BoNT/A binding VHHs were further characterized for their target affinity by surface plasmon resonance (SPR) and their ability to prevent intoxication of primary neurons in culture (Table 1; Figure 1). All VHHs displayed good affinity for their toxin targets with Kd<3 nM. Three VHHs (ciA-B5, -C2 and -H7) proved to be potent toxin neutralizing agents, preventing intoxication of neurons with 10 picomoles (pm) BoNT/A at concentrations near equimolar with toxin. Two VHHs (ciA-D12 and -F12) showed negligible toxin neutralizing activity on primary neurons even at 1000× excess of toxin. Two VHHs (ciA-A5 and -G5), displayed intermediate neutralizing activity. Western and ELISA data (not shown) demonstrated that three anti-BoNT/A VHHs (ciA-H7, -D12 and -F12) recognize the light chain protease domain on the holotoxin. VHH ciA-B5 and -C2 recognize the heavy chain and the other VHHs recognize epitopes apparently requiring both heavy and light chain domains.

**Table 1:** Summary of VHH characterization data.

Clone	Protein	Epitope^A^	Subunit^B^	Neutralization^C^	SPR Kd nM)^D^	Genbank
JDY-33	ciA-H7	A1	Lc	strong	0.04+/−0.03	HQ700708
JDT-2	ciA-D1	A1	Lc	strong	0.71+/−0.004	
JEC-3	ciA-H4	A1	Lc	not done	1.54+/−0.06	
JEC-11	ciA-H11	A1	Lc	not done	4.3+/−0.09	
JDY-46	ciA-C2	A2	Hc-RBD	strong	3.7+/−0.7	HQ700705
JDY-9	ciA-B5	A3	Hc	strong	0.09+/−0.02	HQ700704
JED-27	ciA-F12	A4	Lc	none	0.24+/−0.03	HQ700706
JDU-26	ciA-D12	A5	Lc	none	0.21+/−0.1	HQ700702
JDY-2	ciA-A5	A6	none	weak	1.05+/−0.05	HQ700703
JDY-59	ciA-G5	A7	none	weak	0.32+/−0.03	HQ700707
JFA-10	ciB-H11	B1	none	not done	0.26+/−0.01	HQ700712
JFX-30	ciB-A11	B2	none	not done	0.84+/−0.68	HQ700709
JFV-48	ciB-B5	B3	none	not done	0.97+/−0.04	HQ700711
JFV-40	ciB-B9	B4	none	not done	23+/−5.8	HQ700710
JEZ-2	ciA-H7/B5	A1/A3	not done	strong	0.03+/−0.01	
JFK-21	ciA-F12/D12	A4/A5	not done	not done	0.13+/−0.01	
JGA-3	ciB-A11/B5	B2/B3	not done	not done	5.3+/−1.5	

A VHH epitopes are named arbitrarily based on their inability to compete with the binding of VHHs recognizing other epitopes.

B Subunit recognition was assessed by ELISA with purified BoNT light chain (Lc) or heavy chain (Hc). VHHs recognizing BoNT holotoxin without recognition of purified Lc or Hc are indicated as none. RBD indicates recognition of the 50 kDa carboxyl end receptor binding domain of Hc.

C VHH neutralization was determined by the ability of the VHH to prevent intoxication of primary neurons by 10 pM BoNT/A (Figure 1). ‘Strong’ indicates that the presence of ≤0.1 nM VHH led to obvious toxin neutralization in primary neuron assays (see Figure 1). ‘Weak’ indicates detectable toxin neutralization when the medium contained ≤1 nM VHH. None indicates no toxin neutralization was detected when the medium contained ≤10 nM VHH.

D Surface plasmon resonance (SPR) studies were performed using chips coated with ciBoNTA for BoNT/A VHHs and ciBoNTB for BoNT/B VHHs as described in Methods and Materials.

### Protection from BoNT/A lethality in mice using pools of monomeric anti-BoNT/A VHHs

**Figure 2:**
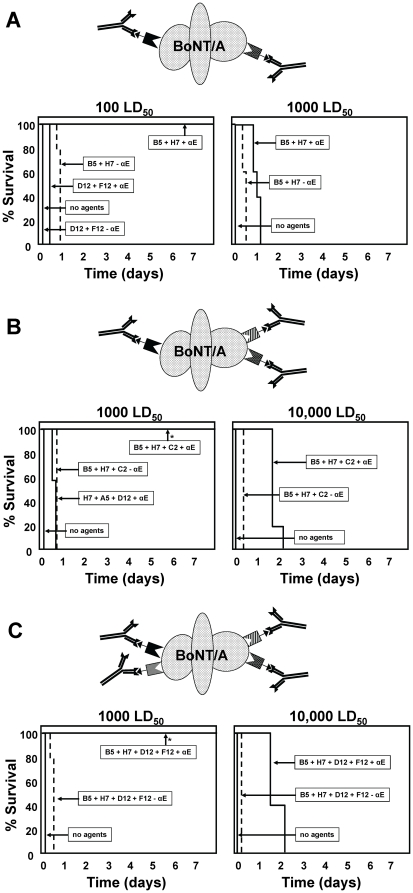
BoNT/A intoxication is prevented in mice by co-administration of a pool of epitopically tagged VHHs and an anti-tag mAb. Symptoms of intoxication and time to death were monitored following administration of the indicated dose of BoNT/A in groups of five mice co-administered with pools of anti-BoNT/A VHHs+/−clearing Ab. The time to death is plotted as % survival as a function of time following administration of the indicated dose of toxin (LD_50_). The pool of ciA-VHHs or control (no agents) that was administered to the mice is indicated by arrows. The presence or absence (dashed lines) of the anti-E-tag clearing Ab (αE) is also indicated. (**A**) Protection from BoNT/A lethality by co-administration of pools containing two different anti-BoNT/A VHH monomers. (**B**) Protection from BoNT/A lethality by co-administration of pools containing three different anti-BoNT/A VHH monomers. (**C**) Protection from BoNT/A lethality by co-administration of pools containing four different anti-BoNT/A VHH monomers. An asterisk indicates that mice did not display any symptoms of intoxication.

The epitopically tagged anti-BoNT/A VHHs were next tested in mice for the ability to prevent toxin induced lethality in the presence or absence of the clearing anti-tag mAb [18]. Various pools of two, three or four different anti-BoNT/A VHHs were co-administered with BoNT/A holotoxin to mice and monitored for symptoms of intoxication and time to death (Figure 2). In all studies employing monomeric VHHs, the total dose of anti-BoNT/A VHH was 2 µg/mouse so only the ‘complexity’ of the VHH pool varied between groups. Mice receiving two anti-BoNT/A VHHs in which both VHHs are unable to neutralize BoNT/A in cell assays (ciA-D12, -F12) did not survive toxin challenge any longer than did control mice receiving no agents (Figure 2A). Even when the anti-E-tag clearing antibody (αE) was included (5 µg), death was only slightly delayed, indicating that serum clearance mediated by the decoration of BoNT/A with two antibodies provides little therapeutic benefit in the absence of toxin neutralization. In contrast, administration of two BoNT/A neutralizing VHHs (ciA-B5, -H7) delayed death from 100 LD_50_ of BoNT/A (∼5 ng) for about a day in the absence of clearing antibody. When clearing antibody was co-administered with the same two neutralizing VHHs, mice fully survived the 100 LD_50_ BoNT/A dose and death was delayed about a day with 1000 LD_50_ BoNT/A. Thus a combination of toxin neutralization and clearance provided greater therapeutic benefit than either protective mechanism could alone. The influence of VHH affinity cannot be excluded in this study (see below) although each VHH had similar sub-nanomolar affinities (Table 1).

Administration of a pool of three different anti-BoNT/A VHHs (ciA-B5, -H7, -C2), each capable of potent toxin neutralization, delayed death less than a day in mice exposed to 1000 LD_50_ (Figure 2B). In the presence of clearing Ab, this pool of tagged VHHs completely protected mice exposed to 1000 LD_50_ of BoNT/A from any apparent symptoms of intoxication and delayed death more than a day in mice exposed to 10,000 LD_50_ (∼0.5 µg of BoNT/A). A different pool of three VHHs in which only one VHH contained potent neutralizing activity was much less effective even in the presence of clearing antibody. These results extend evidence from previously reports [3], [18] that toxin clearance becomes much more effective when BoNT is decorated by at least three Abs and further demonstrates that toxin neutralization can make an important contribution to antitoxin efficacy in this assay.

A pool of four anti-BoNT/A VHHs in which two VHHs were capable of toxin neutralization only slightly delayed death in mice exposed to 1000 LD_50_ BoNT/A (Figure 2C). The same pool administered together with the clearing Ab fully protected from 1000 LD_50_ and delayed death for one or two days in mice exposed to 10,000 LD_50_. In another study, a pool of four anti-BoNT/A VHHs was compared to a pool of six different VHHs. The pool of six contained the same VHHs as the pool of four plus two additional anti-BoNT/A VHHs and was administered in the presence of clearing antibody (Figure S3). The pools of four or six tagged VHHs, administered with anti-tag clearing Ab, both fully protected mice from 1000 LD_50_ and delayed death from 10,000 LD_50_ with almost identical efficacy. Based on these results, and those from several other similar studies (not shown), decoration of BoNT with four Abs improves antitoxin efficacy compared with three Abs while decoration with more antibodies provides little additional potency.

### Role of affinity in antitoxin efficacy

**Figure 3:**
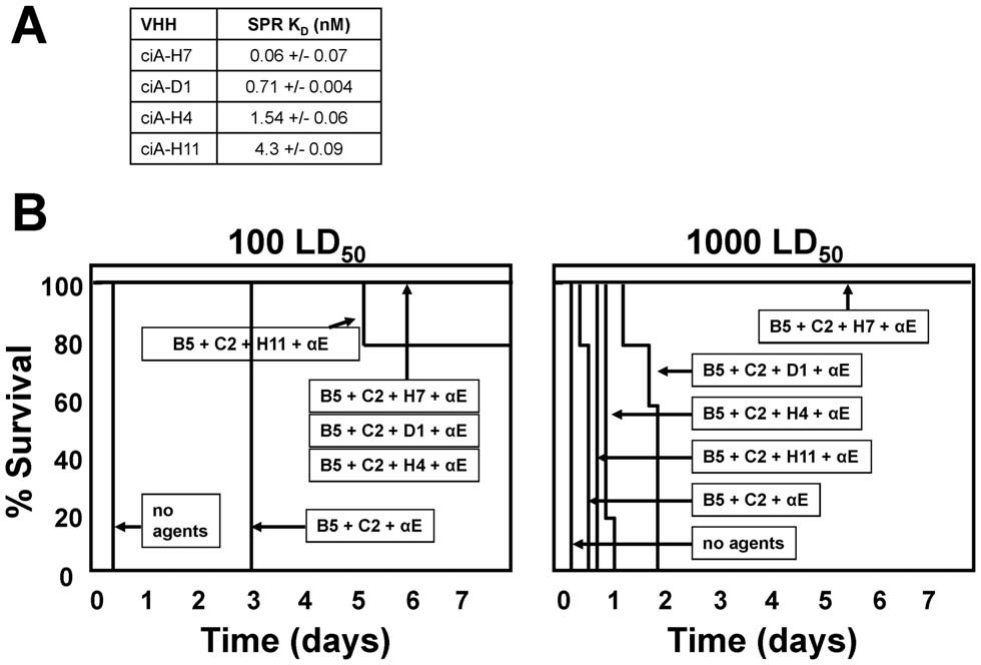
Anti-BoNT/A VHH protection from lethality improves with higher affinity VHHs. (**A**) The K_D_s for four anti-BoNT/A VHHs that each recognize the same epitope (epitope A1; Table 1) based on competition analysis. (**B**) Time to death is plotted as % survival following injection of the indicated dose of BoNT/A in groups of five mice co-administered with pools of different anti-BoNT/A VHHs and clearing Ab. Each VHH pool contained VHHs ciA-B5 and ciA-C2 and one of the four different VHHs recognizing BoNT/A epitope A1. The pool of ciA-VHHs or control (no agents) that were administered to the mice is indicated by arrows.

Both toxin neutralization and clearance mechanisms depend on the binding of antitoxin agents to the toxin. The kinetics of toxin binding (k_on_) and release (k_off_) by the antitoxin binding agents would thus be expected to contribute to their efficacy. To determine the role of toxin affinity to antitoxin efficacy required the identification of multiple VHHs recognizing the same epitope with different affinities. In the course of anti-BoNT/A VHH screening, several VHHs (ciA-D1, H4 and H11) were identified that recognized the same epitope as ciA-H7 based on competition ELISA analysis (sequences shown in Figure S1C). SPR analysis showed that the four VHHs recognized this epitope with affinities ranging from ciA-H7 (K_D_ 0.06 nM) to ciA-H11 (K_D_ 4.3 nM) (Figure 3A). These four VHHs were tested for their efficacy as antitoxin VHHs, each in combination with the two VHHs (ciA-B5, C2) that recognize distinct, non-overlapping epitopes (Figure 3B). The results comparing antitoxin efficacy with 100 and 1000 LD_50_ challenges showed that the antitoxin efficacy of the VHHs was clearly related to their toxin affinities. The highest affinity VHH ciA-H7 was superior to the other VHHs recognizing the same epitope and suggests that sub-nanomolar affinities (K_D_) for the tagged toxin binding agents is necessary to achieve maximal antitoxin efficacy in the mouse lethality assay.

### Antitoxin VHHs expressed as heterodimers

**Figure 4:**
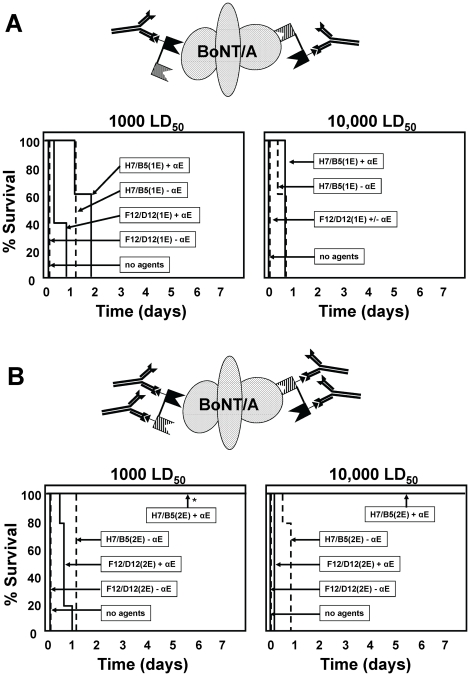
BoNT/A intoxication is prevented in mice by co-administration of a single anti-BoNT/A VHH heterodimer and a clearing Ab. Symptoms of BoNT/A intoxication and lethality were monitored following administration of single- or double-tagged heterodimers of neutralizing or non-neutralizing anti-BoNT/A VHHs+/−clearing Ab, or no agents. Time to death is plotted as % survival as a function of time. (**A**) Heterodimers with a single E-tag consisting of two non-neutralizing VHHs (F12/D12(1E)) or two neutralizing VHHs (H7/B5(1E)) were co-administered to groups of five mice with or without anti-E-tag clearing Ab (αE) and the indicated dose of BoNT/A. (**B**) Heterodimers with two copies of the E-tag and consisting of two non-neutralizing VHHs (F12/D12(1E)) or two neutralizing VHHs (H7/B5(1E)) were co-administered to groups of five mice with or without anti-E-tag clearing Ab (αE) and the indicated dose of BoNT/A. An asterisk indicates that mice did not display any symptoms of intoxication.

Camelid VHHs are stable domains that can be functionally expressed as dimers. Combining two anti-BoNT/A VHHs into a heterodimer would permit one molecule to bind to two different sites on the toxin while likely improving toxin avidity [24]. If the heterodimer contains a single epitopic tag, the one molecule can promote the decoration of BoNT/A with two anti-tag clearing Abs (see diagram in Figure 4A). By addition of a second copy of the epitopic tag to the heterodimer (see diagram in Figure S1D), it should be possible to promote toxin decoration of the toxin with four clearing Abs to yield near maximum clearing efficacy (see diagram in Figure 4B). This hypothesis was tested by preparing two anti-BoNT/A VHH heterodimers in which the two VHHs in the heterodimer were either non-neutralizing (ciA-F12/D12) or potent toxin neutralizing agents (ciA-H7/B5). The two different heterodimers were expressed containing either one or two copies of the epitopic tag (E-tag) (sequences shown in Figure S1E). SPR analysis confirmed that the heterodimer K_D_s are in the range of 10–100 picomolar; significantly higher than the affinities of the component monomers (Table 1).

The antitoxin efficacies of the single-tagged heterodimers in mice (Figure 4) were very similar to those achieved by two corresponding monomers (Figure 2A). The single-tagged heterodimer consisting of non-neutralizing VHHs, ciA-F12/D12(1E), provided no protection from 1000 LD_50_ BoNT/A in the absence of clearing Ab and only slightly delayed death in the presence of clearing Ab. The toxin neutralizing single-tagged heterodimer, ciA-H7/B5(1E), delayed death in mice exposed to 1000 LD_50_ BoNT/A for 1–2 days in the absence of clearing Ab and efficacy was only slightly improved by the addition of clearing Ab. These results are consistent with other data indicating that decoration of toxin with two Abs is not very effective in promoting toxin clearance [3], [18].

Much improved antitoxin efficacy occurred by the simple addition of a second copy of the epitopic tag to the anti-BoNT/A VHH heterodimers when each of these agents was co-administered with clearing Ab. The hypothesis for using a ‘double-tagged heterodimer’ was that the heterodimer VHH would bind at two sites on the toxin and each bound heterodimer would promote toxin decoration with two clearing Abs, thus resulting in decoration of the toxin with four Abs (see diagram in Figure 4B) which was previously shown to be optimal for promoting clearance (see above). A double-tagged heterodimer of non-neutralizing VHHs, ciA-F12/D12(2E), provided virtually no antitoxin efficacy in the absence of clearing Ab as expected for binding agents with little or no toxin neutralizing activity. In the presence of clearing Ab, the same agent fully protected mice from 1000 LD_50_ of BoNT/A and delayed death about a day in mice receiving 10,000 LD_50_ (Figure 4B, Figure S4). Thus the simple addition of a second epitopic tag to the heterodimer dramatically improved the antitoxin efficacy.

In a separate study, the ciA-F12/D12 heterodimer was expressed with one, two or three epitopic tags and tested for antitoxin efficacy in the presence of clearing Ab (Figure S4). The single-tagged heterodimer poorly protected mice from toxin challenge while the double and triple-tagged heterodimers were fully protective to a 100 LD_50_ challenge. There was little improvement in efficacy using the triple-tagged heterodimer as compared to the double-tagged heterodimer, consistent with the prior observation (above) that near maximal clearance is achieved by decorating the target with four antibodies. A titration of the clearing Ab administered with the double-tagged ciA-F12/D12 heterodimer demonstrated that maximal antitoxin efficacy was achieved when the number of Ab molecules administered was approximately equivalent to the number of epitopic tags (Figure S5).

An even more dramatic antitoxin effect was observed using the double-tagged heterodimer, ciA-H7/B5(2E), in which both anti-BoNT/A VHHs possess potent neutralizing activity in cell culture intoxication assays (Figure 1). In the absence of clearing Ab, this agent produced the same antitoxin efficacy as the equivalent single-tagged heterodimer (Figure 4A, B). When clearing Ab was included, the neutralizing double-tagged heterodimer (40 pmoles) became a highly potent antitoxin that fully protected mice from lethality when co-administered with 10,000 LD_50_ BoNT/A (∼1 pmole of holotoxin). A dose-response study was performed in which this agent was co-administered to mice with 1000 LD_50_ (∼0.3 pm) (Figure S6) and demonstrated that 40 pmoles and 13 pmoles completely protected the mice. A dose of 4 pmoles of this neutralizing double-tagged heterodimer had the same protective efficacy for 1000 LD_50_ (Figure S6) as a dose of 40 pmoles did with 10,000 LD_50_ (Figure 4B). These results show that about a 15 fold molar excess of the double-tagged H7/B5 heterodimer binding agent and clearing Ab was sufficient to neutralize and/or clear the vast majority (>99.99%) of BoNT/A when co-administered to a mouse.

### Recombinant antitoxin efficacy in a clinically relevant post-intoxication assay

**Figure 5:**
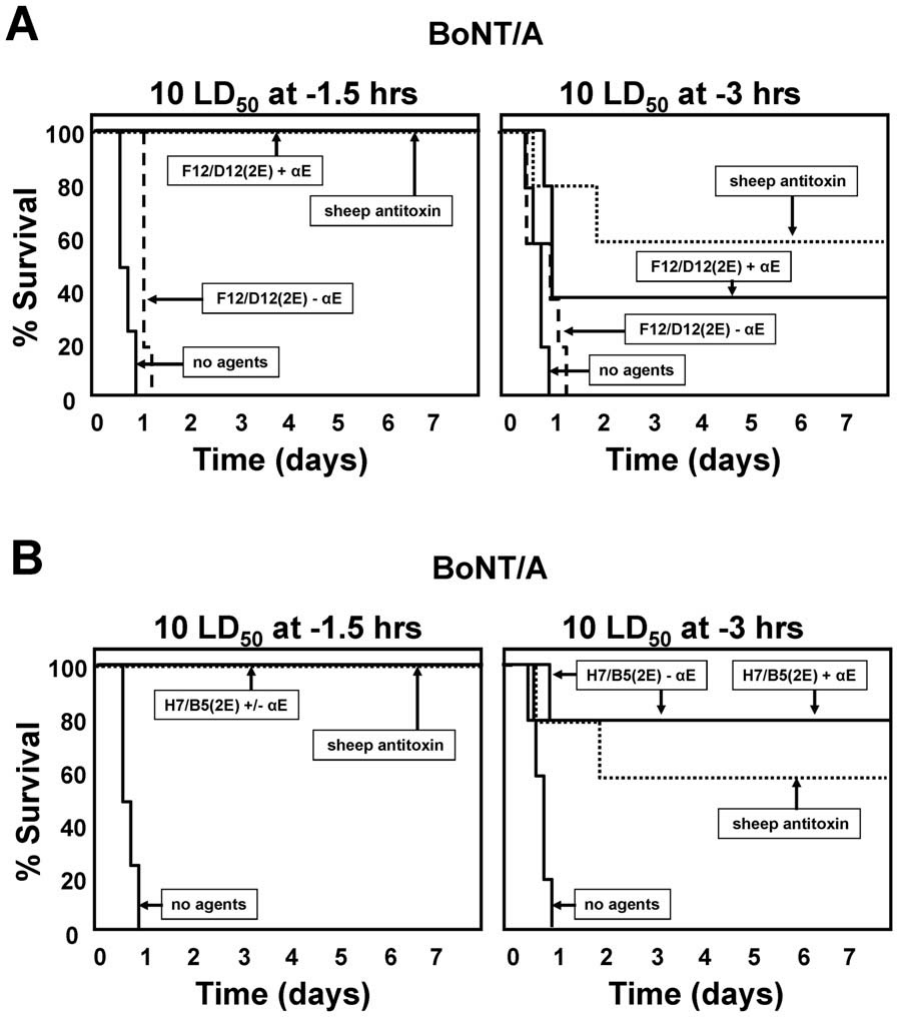
BoNT/A intoxication is prevented by administering a single anti-BoNT/A VHH heterodimer in a clinically-relevant post-intoxication mouse model. A 10 LD_50_ dose of toxin was administered by intraperitoneal injection either 1.5 or 3 hours prior to intravenous administration of antitoxin agents as indicated. Symptoms of BoNT/A intoxication and lethality were monitored following post-intoxication administration of double-tagged heterodimers of neutralizing or non-neutralizing anti-BoNT/A VHHs+/−clearing Ab. The time to death is plotted as % survival as a function of time following administration of toxin. Time to death for mice given no agents or the positive control sheep antitoxin are also shown. (**A**) The double-tagged heterodimer consisted of two non-neutralizing VHHs, ciA-F12 and ciA-D12 (F12/D12(2E)) and was administered with or without anti-E-tag clearing mAb (αE) as indicated. (**B**) The double-tagged heterodimer consisted of two BoNT/A neutralizing VHHs, ciA-H7 and ciA-B5 (H7/B5(2E)) and was administered with or without anti-E-tag clearing mAb (αE) as indicated.

Assays in which varying doses of toxins are co-administered with antitoxin agents permit sensitive quantification of antitoxin efficacy but do not accurately reflect the typical clinical situation. To test antitoxin agents in a more clinically relevant assay, mice were administered with 10 LD_50_ of BoNT/A by intraperitoneal administration and, at various times later, administered intravenously with test agents. As a positive control, we used a potent sheep anti-BoNT/A serum at a dose previously demonstrated to protect 100% of mice from lethality when co-administered with 10,000 LD_50_ of BoNT/A (not shown). Two different anti-BoNT/A VHH heterodimers were tested; the non-neutralizing ciA-F12/D12(2E) heterodimer (Figure 5A) and the neutralizing ciA-H7/B5(2E) heterodimer (Figure 5B). Each heterodimer contained two copies of E-tag and was tested both with and without the anti-E-tag clearing Ab. The non-neutralizing heterodimer had little or no antitoxin efficacy in the absence of clearing Ab, yet when in the presence of this agent it displayed an efficacy nearly equivalent to the positive control sheep antiserum. These results show that toxin clearance alone is sufficient to protect mice from a low dose BoNT challenge (10 LD_50_), even when the agents are administered several hours post-intoxication.

Surprisingly, the neutralizing heterodimer was highly effective as an antitoxin in this assay whether or not clearing Ab was included (Figure 5B). The double-tagged toxin neutralizing heterodimer possessed an antitoxin efficacy equivalent to polyclonal antitoxin even when administered in the absence of anti-tag clearing Ab. These data strongly suggest that BoNT neutralization is sufficient for full antitoxin efficacy when tested in a clinically relevant post-intoxication assay with low dose toxin challenge. The results show that a single protein composed simply of two toxin-neutralizing VHHs has the potential to be as effective as antitoxin sera in clinical situations.

### Antitoxin efficacy of a double-tagged heterodimer targeting BoNT/B

**Figure 6:**
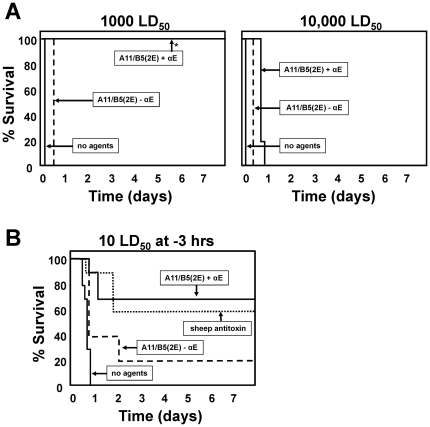
BoNT/B intoxication is prevented by heterodimer antitoxin agents in two models of BoNT/B intoxication in mice. Protection of mice from BoNT/B lethality by administration of a double-tagged heterodimer of anti-BoNT/B VHHs ciB-A11 and ciB-B5 (A11/B5(2E))+/−clearing Ab. Time to death is plotted as % survival as a function of time. An asterisk indicates that mice did not display any symptoms of intoxication. The results shown are combined from two replicate studies. (**A**) Groups of mice were co-administered the indicated LD_50_ dose of BoNT/B together with no agents or the A11/B5(2E) heterodimer VHH with or without anti-E-tag clearing Ab (αE). (**B**) Groups of mice were administered a 10 LD_50_ dose of BoNT/B and three hours later administered no agents, sheep anti-BoNT/B antiserum or the A11/B5(2E) heterodimer VHH with or without anti-E-tag clearing Ab (αE).

The use of double-tagged heterodimer antitoxins was extended to a different toxin target by using VHHs recognizing unique epitopes on BoNT/B holotoxin (Figure S1B). Two of the VHHs, ciB-A11 and B5, were the most potent in vivo in monomer pool studies (not shown) and were selected for expression as a double-tagged heterodimer (ciB-A11/B5(2E)) (sequence in Figure S1E). This agent fully protected mice against 1000 LD_50_ of BoNT/B in the presence of clearing Ab (Figure 6A). In the clinically relevant post-intoxication assay, ciB-A11/B5(2E) was only partially effective in the absence of clearing Ab indicating that the heterodimer is not potent at toxin neutralization. Furthermore, the affinity of this heterodimer for BoNT/B (Kd∼5 nM, Table 1) was weaker than the affinity of either of the two component monomers (Kd∼1 nM each) suggesting that the recombinant heterodimer was not fully functional. Despite this, when the heterodimer was administered with clearing Ab, the treatment was at least as effective as sheep anti-BoNT/B polyclonal antiserum in preventing BoNT/B lethality (Figure 6B). These results demonstrate the efficacy of the heterodimer VHH antitoxin strategy for a second BoNT serotype and suggest the strategy will be effective for treating exposure to the other BoNT serotypes as well as to other pathogenic biomolecules that may occur in patient serum.

## Discussion

This manuscript reports a new approach to the development of antitoxins that employs a single recombinant protein (double-tagged VHH heterodimer) to promote toxin decoration with multiple copies of a single monoclonal antibody (anti-tag mAb) leading to its neutralization and clearance from the body. The approach should have general applicability for clinical situations in which neutralization and/or clearance of a circulating pathogenic biomolecule will result in therapeutic benefit.

Earlier studies had shown that a pool of scFv domain binding agents with specificity for BoNT/A, each containing a common epitopic tag, could direct the decoration of the toxin with multiple anti-tag Abs leading to its clearance via the liver with an efficacy in mouse assays equivalent to conventional polyclonal antitoxin sera [18]. The scFvs served as the toxin targeting agents and the anti-tag mAb served as the clearing agent. Here we show that camelid VHH binding domains, with multiple commercial advantages over scFvs [22], [23], also serve effectively as the toxin targeting agents.

One important advantage of VHHs is the ability to express these agents as heterodimers in which each VHH remains fully functional. This makes possible the fusion of two VHHs that bind to different epitopes on the same toxin target. Incorporation of two epitope tags on the heterodimer permits decoration of the toxin with two clearing Abs at each epitope, a total of four mAbs on the toxin to promote efficient toxin clearance. Since each double-tagged heterodimeric binding agent binds to only two mAbs, the agent itself should not be effectively cleared by low affinity Fc receptors unless bound to toxin. The heterodimers also proved to have much greater apparent affinity for toxin than the equivalent pool of two monomers and this likely contributed to the substantial gains in antitoxin efficacy achieved with the heterodimer antitoxins in mouse models of BoNT intoxication.

The ability of antitoxin antibodies to protect animals from the symptoms of toxin exposure can be influenced by several factors. The dose of antitoxin agent and the timing of antitoxin administration relative to exposure to toxin are obvious factors that influence efficacy. In addition, the affinity of the antibodies for the toxin will influence the ability of the antibody to bind (k_on_) and remain bound (k_off_) to the toxin and exert its effect. The further ability of the antibody to inhibit the enzymatic activity of the toxin and/or prevent its entry into target cells (i.e. neutralization) also must be expected to play a key role. Finally, the ability of the antibodies to promote the clearance of the toxin from the serum will permit the antitoxin to reduce the pool of toxin in circulation. Little is known about the relative importance neutralization vs clearance mechanisms to antitoxin efficacy.

In this study, insight into antitoxin mechanism was possible since experiments were performed that separately tested the roles of toxin neutralization and clearance in determining antitoxin efficacy. The role of neutralization could be assessed by comparing the efficacy of VHHs that are non-neutralizing with toxin neutralizing agents. The role of clearance could be analyzed by comparing antitoxin efficacy of identical non-neutralizing VHH agents in the presence or absence of the anti-tag clearing antibody. The results clearly demonstrate that agents possessing potent BoNT neutralizing activity can be very effective in mouse intoxication models in the absence of clearance. Similarly, agents that have little or no antitoxin efficacy in the absence of clearing Ab can become highly effective simply by co-administration of clearing Ab to promote toxin clearance. Furthermore, our results indicate that combining neutralization and clearance is essential for maximal antitoxin efficacy when the toxin doses are very high. Interestingly, when low BoNT doses were employed, such as the 10 LD_50_ dose used in the clinically-relevant post-intoxication model, toxin clearance or neutralization alone were each sufficient and about equally effective. Presumably this occurs because clearance or neutralization alone are each capable of sufficiently reducing the level of active BoNT when the antitoxin is administered during the ‘window of opportunity’ [25] that exists between exposure and the onset of irreversible symptoms. At much higher BoNT doses, both toxin clearance and neutralization appear necessary to permit survival.

The toxins employed in this study were *Botulinum* neurotoxins (BoNTs) and additional studies will be necessary to assess the extent to which the heterodimer binding agent antitoxin strategy described here will prove efficacious for other toxins. BoNTs are extremely potent with exquisite specificity for neurons and normally remain in circulation until they enter a neuron or are naturally cleared. Because of the high potency of BoNT, measurable toxicity occurs with extremely small amounts of circulating toxin. With less potent toxins, intoxication requires higher doses of toxin and thus higher concentrations of Ab are required for antitoxin efficacy.

For toxins with less target cell specificity, such as those from *Clostridium difficile* and *Escherichia coli* or ricin, the toxins are likely to spend shorter time in circulation and, in these cases, toxin neutralization may be more important to efficacy than toxin clearance. Where the toxins are active on cells with low affinity Fc receptors involved in toxin clearance, antitoxins that promote clearance may not be of benefit. For these reasons, it is difficult to predict whether the most effective heterodimer antitoxin strategy should promote toxin clearance or focus entirely on toxin neutralization.

One concern with the use of heterodimer binding agents is the possibility that the binding agents will be immunogenic and elicit an immune response that reduces or eliminates the therapeutic efficacy. VHHs are not considered to be strongly immunogenic and the immunogenicity can by reduced further by introducing targeted mutations [26]. Alternative non-Ab binding agents such as DARPins, Anticalins or AdNectins [27] should be able to replace VHHs if sufficient target affinity can be achieved and these are specifically designed to be poor immunogens. Some immunogenicity may be tolerable and may even improve efficacy by promoting target clearance.

Studies reported here have demonstrated that a single heterodimer protein composed of two distinct toxin neutralizing VHHs has efficacy equivalent to polyclonal antitoxin serum in a clinically-relevant post-intoxication BoNT lethality assay. The ability to prevent intoxication with a single polypeptide substantially simplifies the commercial production of the antitoxin and makes possible genetic delivery approaches such as with DNA or viral vectors. Improved therapeutic efficacy is possible by promoting the clearance of the pathogenic biomolecule target and this can be achieved by producing the heterodimer with two copies of an epitopic tag and co-administering the agent with an anti-tag clearing mAb. This results in the decoration of the target with up to four mAbs which leads to clearance presumably by a low affinity FcR-dependent pathway. The clearing mAb itself could be made unnecessary by producing the heterodimer fused to a peptide or VHH that binds to low-affinity FcR. Using these varied strategies, it should be possible to develop new and versatile therapeutic approaches that permit the neutralization and/or clearance of one or more targeted pathogenic biomolecules from the circulation of patients.

## Materials and Methods

### Ethics statement

All studies were carried out in strict accordance with the recommendations delineated in the Guide for the Care and Use of Laboratory Animals of the National Institutes of Health. The procedures used were approved by the Tufts University Institutional Animal Care and Use Committee (IACUC) and were performed under Protocols #G2010-60 and G874-07.

### Toxins and reagents

Botulinum neurotoxin serotype A1 (BoNT/A) and serotype B (BoNT/B) were obtained from Metabiologics Inc. Each batch of toxin was assayed to establish LD_50_ dose. BoNT complex was used for animal studies and BoNT holotoxin was used for the cell-based studies. Purified recombinant BoNT serotype A1 and B holotoxins containing mutations rendering them catalytically inactive (ciBoNTA, ciBoNTB) were produced as previously described [28]. Sheep anti-BoNT/A1 antiserum was produced by immunization of sheep with BoNT/A1 toxoid followed by BoNT/A1 holotoxin. Less than 1 µl of this sheep antitoxin serum protects mice from lethality when co-administered with 10,000 LD_50_ of BoNT/A1. Reagents for Western blotting were purchased from KPL. Antibodies used were rabbit anti-SNAP25 antibody (Sigma); goat anti-rabbit HRP antiserum (Sigma); anti-E-tag mAb (Phadia); HRP anti-E-tag mAb (GE Healthcare). All studies with holotoxin were performed within a Select Agent laboratory registered with the CDC.

### Alpaca immunization and VHH-display library preparation

Two alpacas were immunized with ciBoNTA and two with ciBoNTB. The immunization regimen employed 100 µg of protein in the primary immunization and 50 µg in three subsequent boosting immunizations at about 3 weekly intervals in alum/CpG adjuvant. Five days following the final boost, blood was obtained for lymphocyte preparation and VHH-display phage libraries were prepared from the immunized alpacas as previously described [29], [30]. More than 10^6^ independent clones were prepared from B cells of alpacas successfully immunized with each of the BoNT immunogens.

### Anti-BoNT VHH identification and preparation

The VHH-display phage libraries were panned for binding to ciBoNTA or ciBoNTB targets that were coated onto a well of a 12 well plate. Coating was performed by overnight incubation with one ml of a 5 µg/ml target solution in PBS at 4°C followed by washing with PBS and 2 hrs incubation at 37°C with blocking agent (4% non-fat dried milk powder in PBS). Panning, phage recovery and clone fingerprinting were performed as previously described [29], [30]. A total of 192 and 142 VHH clones were identified as strong positives for binding to BoNT/A and BoNT/B respectively based on phage ELISA signals. Of the strong positives, 62 unique DNA fingerprints were identified among the VHHs selected for binding to BoNT/A and 32 for VHHs selected for binding to BoNT/B. DNA sequences of the VHH coding regions was obtained for each phage clone and compared for homologies. Based on this analysis, 12 of the anti-BoNT/A VHHs and 11 anti-BoNT/B were identified as unlikely to have common B cell clonal origins and selected for protein expression.

Expression and purification of VHHs in *E. coli* as recombinant thioredoxin (Trx) fusion proteins containing hexahistidine was performed as previously described [30]. For heterodimers, DNA encoding two different VHHs were joined in frame downstream of Trx and separated by DNA encoding a 15 amino acid flexible spacer ((GGGGS)_3_). All VHHs were expressed with a carboxyl terminal E-tag epitope. Some expression constructions were engineered to contain a second copy of the E-tag by introducing the coding DNA in frame between the Trx and VHH domains. An example of a Trx fusion to a VHH heterodimer with two E-tags is shown in Figure S1C. A third E-tag was introduced in frame within the DNA encoding the flexible spacer of the heterodimer containing ciA-D12 and ciA-F12 to create a triple tagged heterodimer (D12/F12(3E)).

### VHH target binding competition analysis

Phage displaying individual VHHs were prepared and titered by phage dilution ELISA [29] for recognition of ciBoNTA or ciBoNTB using HRP/anti-M13 Ab for detection. A dilution was selected for each phage preparation that produced a signal near the top of the linear range of the ELISA signal. The selected phage dilution (100 µl) for each VHH-displayed phage preparation were added to a 96 well plate that has been coated with ciBoNTA or ciBoNTB and then pre-incubated for 30 minutes with 100 µl of a 10 µg/ml solution containing a purified Trx/VHH fusion protein test agent or control in PBS. After an hour, the wells were washed and phage binding was detected as above. Test VHHs that reduced target binding of phage-displayed VHHs by less than two fold vs controls were considered to recognize distinct epitopes. Positive controls were performed in which the Trx/VHH competitor contained the same VHH as displayed on phage and typically reduced the ELISA signal >95%.

### Characterization of VHH binding properties

VHHs were tested for binding to native or atoxic mutant BoNT holotoxins by standard ELISA using plates coated with 100 µl of 1 µg/ml protein. VHHs were also tested for recognition of BoNT subunits by a dilution series ELISA (10 µg/ml, 1∶5 dilutions) using plates coated by overnight incubation at 4°C with 5 µg/ml purified recombinant BoNT light chain [30] or 1 µg/ml BoNT heavy chain. VHH binding was detected with HRP-anti-E-tag mAb (GE Healthcare). VHHs were also characterized for recognition of subunits by Western blotting on BoNT holotoxin following standard SDS-PAGE (4–20% gel) with samples boiled in SDS sample buffer under reducing conditions (5% βME). VHHs were incubated with filters at 10 µg/ml and bound VHH detected with HRP-anti-E-tag mAb (GE Healthcare) by standard procedures.

### Kinetic analysis by surface plasmon resonance

Studies to assess the kinetic parameters of the VHHs were performed using a ProteOn XPR36 Protein Interaction Array System (Bio-Rad, Hercules, CA) after immobilization of ciBoNTA or ciBoNTB by amine coupling chemistry using the manufacturer recommended protocol. Briefly, after activation of a GLH (high protein immobilization capacity) chip surface with a mixture of 0.4 M ethyl(dimethylaminopropyl) carbodiimide (EDC) and 0.1 M N-hydroxysulfosuccinimide (sulfo-NHS) injected for 300 s at 30 µL/min, ciBoNTA or ciBoNTB was immobilized by passing a 60 µg/mL solution of the protein at pH 5 over the surface for 180 s at 25 µL/min. The surface was deactivated with a 30 µL/min injection of 1 M ethanolamine for 300 s. A concentration series for each VHH (between 2.5 nM and 1000 nM, optimized for each antibody fragment) was passed over the surface at 100 µL/min for 60 s, then dissociation was recorded for 600 s or 1200 s. The surface was then regenerated with a 36 s injection of 10 mM glycine, pH 2.0 at 50 µL/min. Running buffer for these studies was 10 mM Hepes, pH 7.4, 150 mM NaCl, 0.005% Tween-20. Data was evaluated with ProteOn Manager software (version 2.1.2) using the Langmuir interaction model.

### BoNT neutralization assay using primary neurons

Neuronal granule cells from the pooled cerebella of either 7–8 day old Sprague-Dawley rats or 5–7 day old CD-1 mice were harvested as described by Skaper et al [31] and cultured in 24 well plates as described by Eubanks et al [32]. After at least a week of culture the well volumes were adjusted to 0.5 ml containing various VHH dilutions or buffer controls followed immediately by addition of BoNT/A in 0.5 ml to a final 10 pM. After overnight at 37°C, cells were harvested and the extent of SNAP25 cleavage assessed by Western blot as previously described [32].

### Standard mouse toxin lethality assay

Female CD1 mice 15–17 g each (Charles River Labs) were received 5 days prior to use. One day prior to initiation of study, mice were weighed and placed into groups in an effort to minimize inter-group weight variation. Appropriate dilutions of the test agents were prepared in PBS. BoNT holotoxins were separately prepared in PBS+0.2% gelatin (Sigma) at the desired doses. 600 µl of test agent and 600 µl of the toxin were combined and incubated at room temperature for 30 minutes. 200 µl of the mixture was administered by intravenous injection at time 0 to mice in groups of five. Mice were monitored at least four times per day and scored for overall disposition, severity of abdominal breathing, presence of open-mouth breathing, activity level, presence of lethargy, and mortality. Moribund mice were euthanized. Time to onset of symptoms and time to death were established for each mouse [33].

### Mouse toxin lethality assay with agents administered post-intoxication

This assay is a modification of an assay developed by Cheng et al [25]. Groups of mice were prepared as above. Mice were administered 10 LD_50_ of BoNT/A by intraperitoneal injection. At indicated times post-intoxication, mice were administered 200 ul of test agent in PBS by intravenous injection. Mice were monitored for symptoms of intoxication as above.

## Supporting Information

Figure S1 **Protein sequences of anti-BoNT/A and anti-BoNT/B VHH monomers and heterodimers.** (**A**) Protein sequences of VHHs recognizing unique epitopes on BoNT/A (ciA) are shown aligned for homology. Regions represented by dashes are gaps. (**B**) Protein sequences of VHHs recognizing unique epitopes on BoNT/B (ciB) are shown aligned for homology. Regions represented by dashes are gaps. (**C**) Protein sequences of three VHHs recognizing the same BoNT/A epitope as ciA-H7 are shown aligned for homology. VHHs in A and B also contain Q(L/V)QLVE at the amino end that is encoded by the PCR primer used to generate the VHH-display library [34]. The eight amino acids shown at the carboxyl end are encoded by either the short hinge or long hinge PCR primer that were used to generate the library [34]. (**D**) Schematic diagram of the domain structure of a double-tagged VHH heterodimer protein. Proteins were expressed in pET32b with an amino-terminal E. coli thioredoxin. Domain abbreviations used were: Trx, *E. coli* thioredoxin; 6H, hexahistidine domain including enterokinase cleavage site (DDDDK); E, E-tag peptide; VHH-1, first VHH; fs, flexible spacer domain ((GGGGS)_5_); VHH-2 second VHH. Relative domain sizes in the diagram are approximate. (**E**) Protein sequences of the entire translation product of three recombinant VHH heterodimers containing two copies of E-tag. The E-tag sequences (GAPVPYPDPLEPR) are underlined. The amino acid sequences preceding the first E-tag in each protein contains the thioredoxin fusion partner and hexahistidine encoded by the pET32b expression vector. The VHH sequences are flanked by the two E-tag peptides and separated by the unstructured spacer ((GGGGS)_3_).
(TIF)

Figure S2 **SDS-PAGE analysis of purified VHH monomers and heterodimers.** Following gel electrophoresis of 1 µg of the indicated purified proteins, gels were stained for protein. (**A**) VHH monomers recognizing BoNT/A (ciA-). (**B**) VHH heterodimers recognizing BoNT/A (ciA-) or BoNT/B (ciB-) in which the two indicated VHHs are expressed with the first VHH at the amino end and the second VHH at the carboxyl end. An E indicates the presence and position of peptide E-tags relative to the VHHs. The migration positions of molecular weight markers are shown with arrows.
(TIF)

Figure S3 **Time to death plots following co-injection BoNT/A and pools of four or six anti-BoNT/A VHHs+clearing Ab (αE).** The contents of the pool of ciA-VHHs or control (no agents) that was administered to the mice is indicated by arrows. An asterisk indicates that mice did not display any symptoms of intoxication.
(TIF)

Figure S4 **Antitoxin efficacy of non-neutralizing anti-BoNT/A VHH heterodimer ciA-F12/D12 containing one, two or three E-tag epitopes when co-administered with anti-E-tag clearing Ab and BoNT/A.** The % survival is plotted as a function of time for groups of five mice. Groups of mice were administered 20 pmoles of the heterodimer of VHHs ciA-F12 and ciA-D12 (F12/D12) containing one (1E), two (2E) or three (3E) copies of the E-tag epitope as indicated by arrows. Another group of mice received a pool of the two monomer VHHs (20 pm each), ciA-F12 and ciA-D12. The toxin dose is indicated in LD_50_. All mice received 60 pm of anti-E-tag clearing Ab (αE).
(TIF)

Figure S5 **Antitoxin efficacy of non-neutralizing anti-BoNT/A VHH heterodimer ciA-F12/D12 containing two copies of E-tag and co-administered with BoNT/A and varying doses of anti-E-tag clearing Ab.** The % survival is plotted as a function of time for groups of five mice. Groups of mice were co-administered BoNT/A, 20 pmoles of the non-neutralizing VHH heterodimer ciA-F12/D12 containing two copies of E-tag (F12/D12(2E)) or no agents and anti-E-tag mAb as the indicated dose. The toxin dose is indicated in LD_50_.
(TIF)

Figure S6 **Titration of the BoNT/A antitoxin efficacy of the neutralizing anti-BoNT/A VHH heterodimer co-administered with clearing Ab.** The % survival is plotted as a function of time for groups of five mice. Groups of mice were administered 1000 LD_50_ of BoNT/A (∼0.3 pmoles) and either no agents or 40, 13, 4.4 or 1.5 pmoles of the double-tagged BoNT/A neutralizing VHH heterodimer, ciA-H7/B5(2E).
(TIF)
