# Supplementary figures and images for: A Novel Strategy for Development of Recombinant Antitoxin Therapeutics Tested in a Mouse Botulism Model
Source: PLoS One. 2012 Jan 6;7(1):e29941. doi: 10.1371/journal.pone.0029941 (PMC3253120; doi:10.1371/journal.pone.0029941)

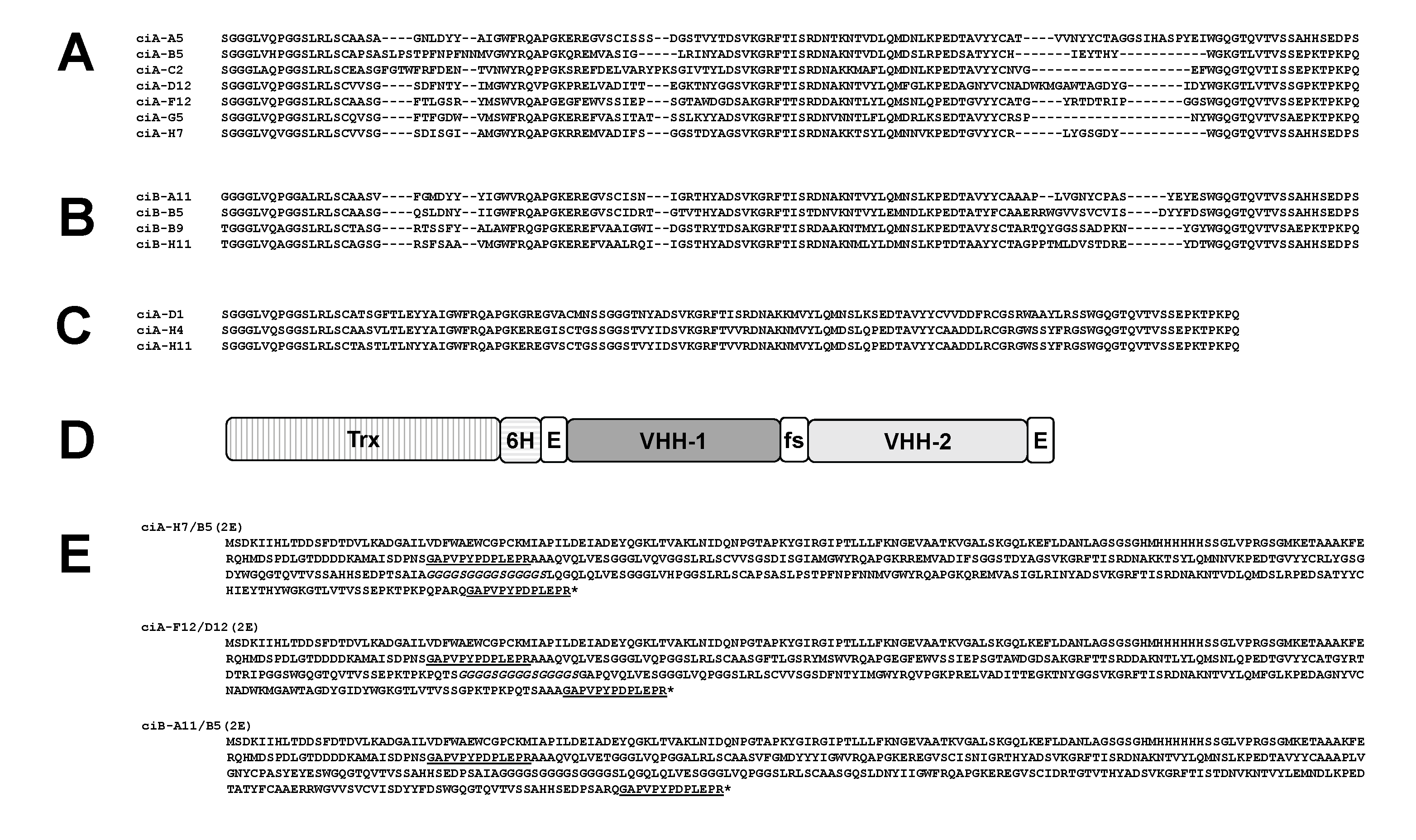

Supplement: Figure S1 — Protein sequences of anti-BoNT/A and anti-BoNT/B VHH monomers and heterodimers. (A) Protein sequences of VHHs recognizing unique epitopes on BoNT/A (ciA) are shown aligned for homology. Regions represented by dashes are gaps. (B) Protein sequences of VHHs recognizing unique epitopes on BoNT/B (ciB) are shown aligned for homology. Regions represented by dashes are gaps. (C) Protein sequences of three VHHs recognizing the same BoNT/A epitope as ciA-H7 are shown aligned for homology. VHHs in A and B also contain Q(L/V)QLVE at the amino end that is encoded by the PCR primer used to generate the VHH-display library [34]. The eight amino acids shown at the carboxyl end are encoded by either the short hinge or long hinge PCR primer that were used to generate the library [34]. (D) Schematic diagram of the domain structure of a double-tagged VHH heterodimer protein. Proteins were expressed in pET32b with an amino-terminal E. coli thioredoxin. Domain abbreviations used were: Trx, E. coli thioredoxin; 6H, hexahistidine domain including enterokinase cleavage site (DDDDK); E, E-tag peptide; VHH-1, first VHH; fs, flexible spacer domain ((GGGGS)5); VHH-2 second VHH. Relative domain sizes in the diagram are approximate. (E) Protein sequences of the entire translation product of three recombinant VHH heterodimers containing two copies of E-tag. The E-tag sequences (GAPVPYPDPLEPR) are underlined. The amino acid sequences preceding the first E-tag in each protein contains the thioredoxin fusion partner and hexahistidine encoded by the pET32b expression vector. The VHH sequences are flanked by the two E-tag peptides and separated by the unstructured spacer ((GGGGS)3). (TIF) [file pone.0029941.s001.tif]

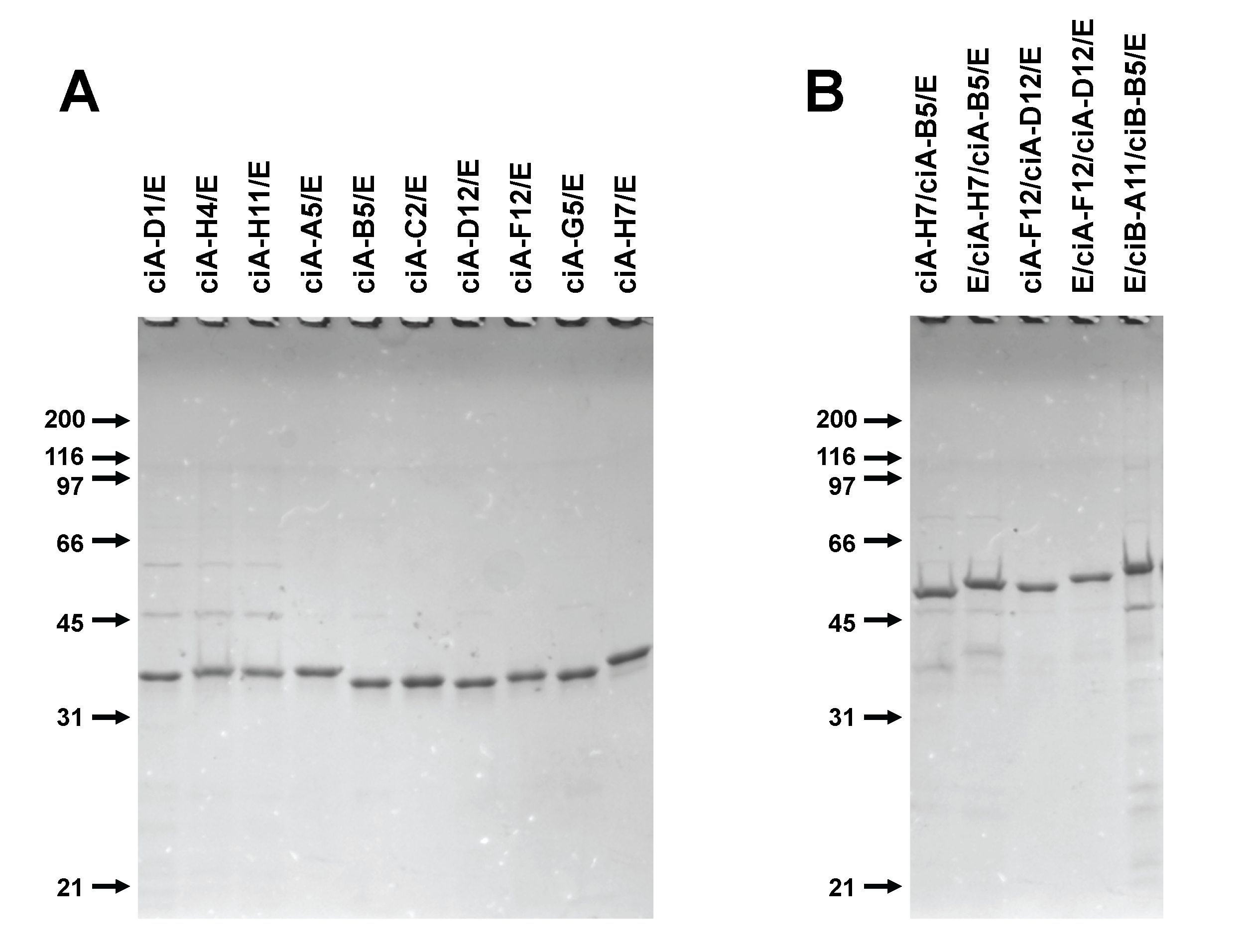

Supplement: Figure S2 — SDS-PAGE analysis of purified VHH monomers and heterodimers. Following gel electrophoresis of 1 µg of the indicated purified proteins, gels were stained for protein. (A) VHH monomers recognizing BoNT/A (ciA-). (B) VHH heterodimers recognizing BoNT/A (ciA-) or BoNT/B (ciB-) in which the two indicated VHHs are expressed with the first VHH at the amino end and the second VHH at the carboxyl end. An E indicates the presence and position of peptide E-tags relative to the VHHs. The migration positions of molecular weight markers are shown with arrows. (TIF) [file pone.0029941.s002.tif]

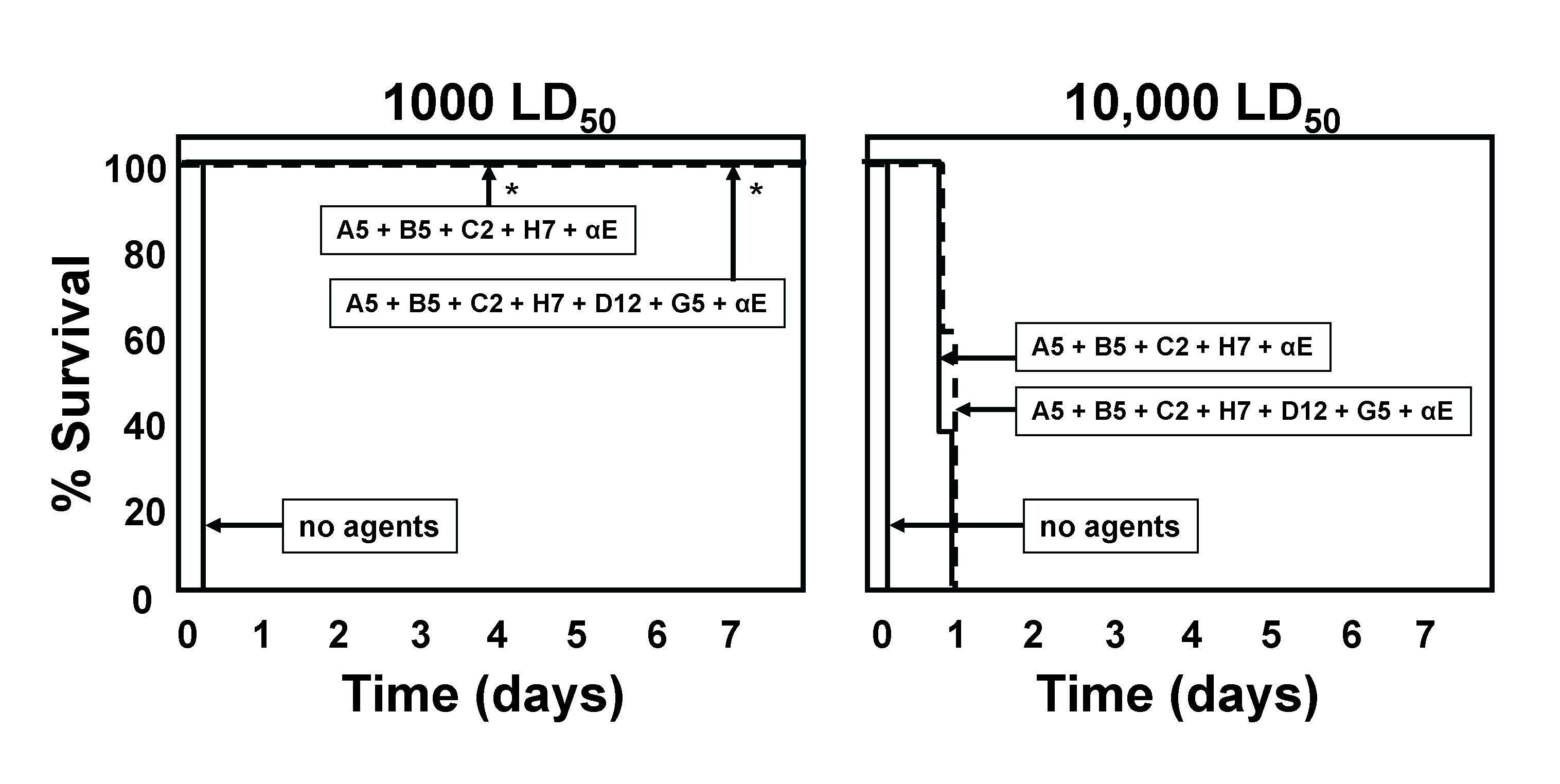

Supplement: Figure S3 — Time to death plots following co-injection BoNT/A and pools of four or six anti-BoNT/A VHHs+clearing Ab (αE). The contents of the pool of ciA-VHHs or control (no agents) that was administered to the mice is indicated by arrows. An asterisk indicates that mice did not display any symptoms of intoxication. (TIF) [file pone.0029941.s003.tif]

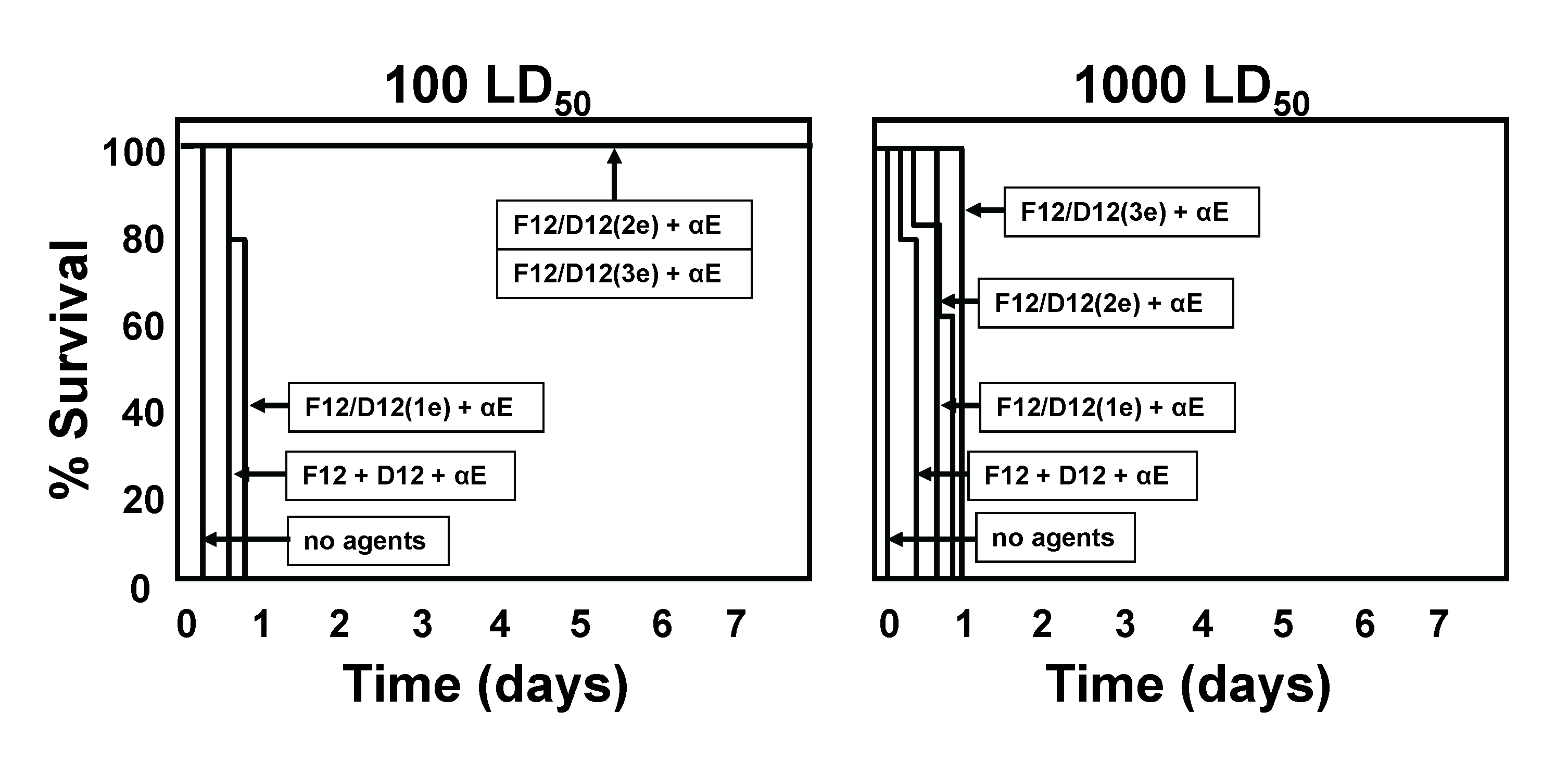

Supplement: Figure S4 — Antitoxin efficacy of non-neutralizing anti-BoNT/A VHH heterodimer ciA-F12/D12 containing one, two or three E-tag epitopes when co-administered with anti-E-tag clearing Ab and BoNT/A. The % survival is plotted as a function of time for groups of five mice. Groups of mice were administered 20 pmoles of the heterodimer of VHHs ciA-F12 and ciA-D12 (F12/D12) containing one (1E), two (2E) or three (3E) copies of the E-tag epitope as indicated by arrows. Another group of mice received a pool of the two monomer VHHs (20 pm each), ciA-F12 and ciA-D12. The toxin dose is indicated in LD50. All mice received 60 pm of anti-E-tag clearing Ab (αE). (TIF) [file pone.0029941.s004.tif]

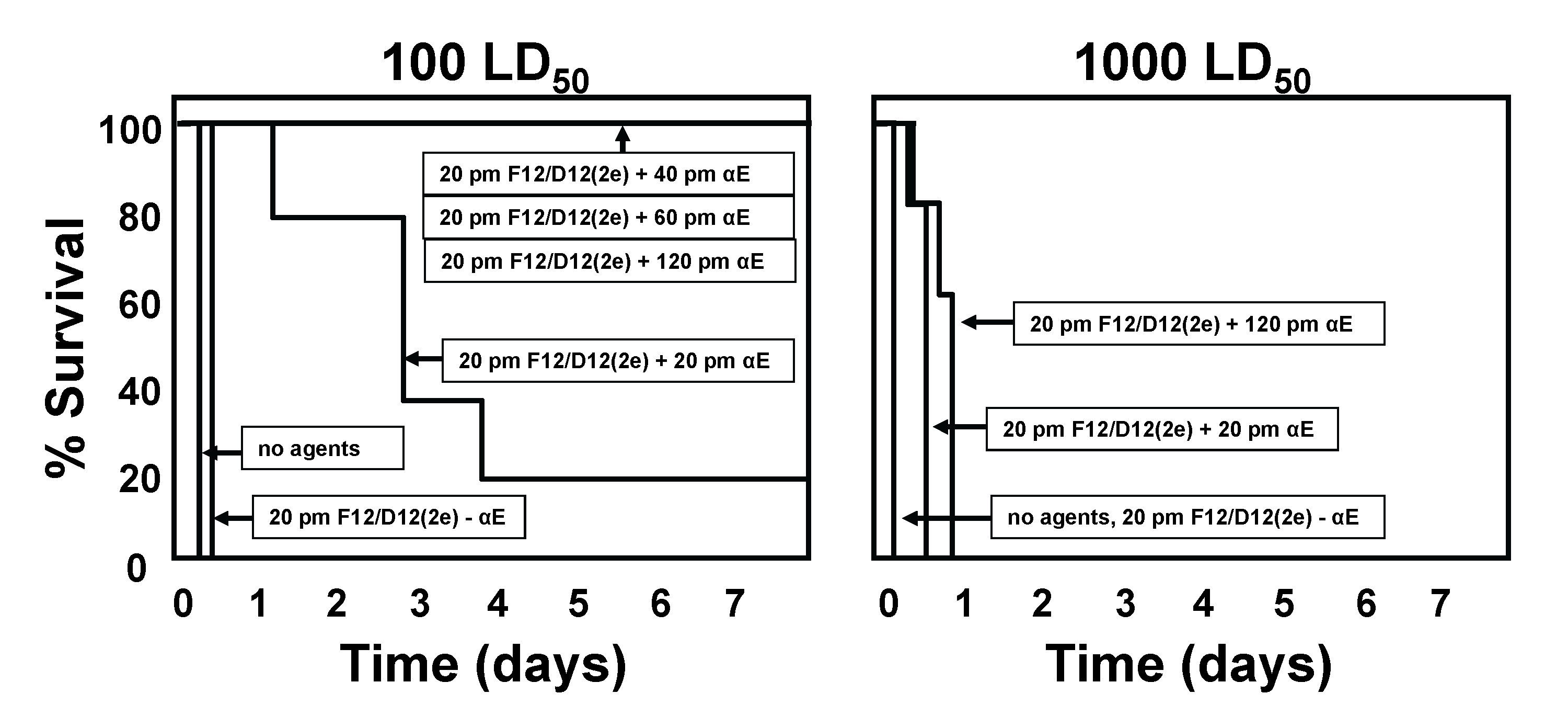

Supplement: Figure S5 — Antitoxin efficacy of non-neutralizing anti-BoNT/A VHH heterodimer ciA-F12/D12 containing two copies of E-tag and co-administered with BoNT/A and varying doses of anti-E-tag clearing Ab. The % survival is plotted as a function of time for groups of five mice. Groups of mice were co-administered BoNT/A, 20 pmoles of the non-neutralizing VHH heterodimer ciA-F12/D12 containing two copies of E-tag (F12/D12(2E)) or no agents and anti-E-tag mAb as the indicated dose. The toxin dose is indicated in LD50. (TIF) [file pone.0029941.s005.tif]

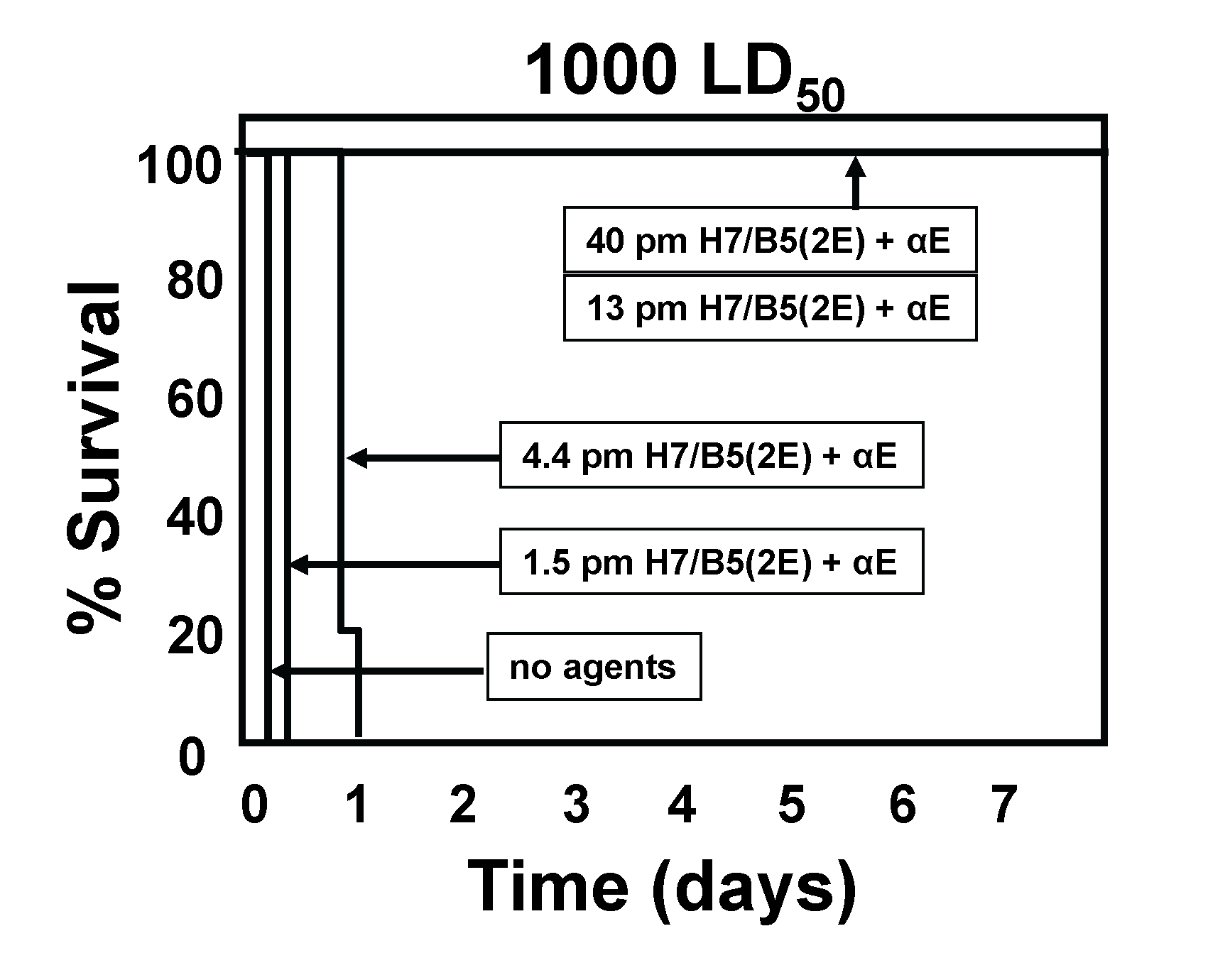

Supplement: Figure S6 — Titration of the BoNT/A antitoxin efficacy of the neutralizing anti-BoNT/A VHH heterodimer co-administered with clearing Ab. The % survival is plotted as a function of time for groups of five mice. Groups of mice were administered 1000 LD50 of BoNT/A (∼0.3 pmoles) and either no agents or 40, 13, 4.4 or 1.5 pmoles of the double-tagged BoNT/A neutralizing VHH heterodimer, ciA-H7/B5(2E). (TIF) [file pone.0029941.s006.tif]
